# Immune reconstitution inflammatory syndrome due to *Mycobacterium avium* complex successfully followed up using ^18^ F-fluorodeoxyglucose positron emission tomography-computed tomography in a patient with human immunodeficiency virus infection: A case report

**DOI:** 10.1186/s12880-015-0063-2

**Published:** 2015-07-18

**Authors:** Ho Namkoong, Hiroshi Fujiwara, Makoto Ishii, Kazuma Yagi, Mizuha Haraguchi, Masako Matsusaka, Shoji Suzuki, Takanori Asakura, Takahiro Asami, Fumitake Saito, Koichi Fukunaga, Sadatomo Tasaka, Tomoko Betsuyaku, Naoki Hasegawa

**Affiliations:** Division of Pulmonary Medicine, Department of Medicine, Keio University School of Medicine, 35 Shinanomachi, Shinjuku-ku, Tokyo 160-8582 Japan; Japan Society for the Promotion of Science, Tokyo, Japan; Center for Infectious Diseases and Infection Control, Keio University School of Medicine, Tokyo, Japan

**Keywords:** Immune reconstitution inflammatory syndrome, Human immunodeficiency virus, Nontuberculous mycobacteria, *Mycobacterium avium* complex, ^18^ F-fluorodeoxyglucose positron emission tomography/computed tomography

## Abstract

**Background:**

In human immunodeficiency virus (HIV)-infected patients, immune reconstitution inflammatory syndrome (IRIS) due to nontuberculous mycobacteria (NTM) infection is one of the most difficult types of IRIS to manage. ^18^ F-fluorodeoxyglucose positron emission tomography/computed tomography (^18^ F-FDG PET/CT) has been suggested as a useful tool for evaluating the inflammatory status of HIV-infected patients. We present the first case of *Mycobacterium avium* complex (MAC)-associated IRIS (MAC-IRIS) that was successfully followed up using ^18^ F-FDG PET/CT.

**Case presentation:**

A 44-year-old homosexual Japanese man was referred to our hospital with fever and dyspnea. He was diagnosed with *Pneumocystis jiroveci* pneumonia and found to be HIV positive. After the initiation of combined antiretroviral therapy (cART), the patient’s mediastinal and bilateral hilar lymphadenopathy gradually enlarged, and bilateral infiltrates appeared in the upper lung fields. ^18^ F-FDG PET/CT was performed five months after the initiation of cART and showed intense accumulation of fluorodeoxyglucose (FDG) corresponding to the lesions of infiltration as well as the mediastinal and bilateral hilar lymphadenopathy. A bronchial wash culture and pathology findings led to a diagnosis of MAC-IRIS. Anti-mycobacterial chemotherapy with rifampicin, ethambutol, clarithromycin, and levofloxacin was started. One year after the chemotherapy was initiated, there was a significant reduction in FDG uptake in the area of the lesions except in the mediastinal lymph node. This implied incomplete resolution of the MAC-IRIS-related inflammation. Anti-mycobacterial chemotherapy was continued because of the residual lesion. To date, the patient has not experienced a recurrence of MAC-IRIS, a period of nine months.

**Conclusion:**

We present a case of MAC-IRIS in an HIV-infected patient whose disease activity was successfully followed up using ^18^ F-FDG PET/CT. Our data suggest that ^18^ F-FDG PET/CT is useful for evaluating the disease activity of NTM-IRIS and assessing the appropriate duration of anti-mycobacterial chemotherapy for NTM-IRIS in HIV-infected patients.

## Background

Combined antiretroviral therapy (cART) targeting the human immunodeficiency virus (HIV) has dramatically improved the prognosis of HIV-infected patients by suppressing HIV and restoring the disrupted host immune system [1]. During host immune recovery after the initiation of cART, a subset of HIV-infected patients experience a paradoxical worsening of coexisting infections or the appearance of new diseases; this has been named the immune reconstitution inflammatory syndrome (IRIS). Since IRIS, a fatal complication, occurs in 13 % of HIV-infected patients, the clinical importance of these patients has increased in the cART era [2]. IRIS is associated with a variety of medical conditions including cytomegalovirus retinitis, cryptococcal meningitis, tuberculosis (TB), progressive multifocal leukoencephalopathy, and herpes zoster infection [2]. Among these IRIS conditions, nontuberculous mycobacteria (NTM)-associated IRIS (NTM-IRIS) is one of the most difficult diseases to manage since it often results in a poor prognosis, even if intensive anti-NTM chemotherapy is initiated [3]. A detailed clinical characterization of NTM-IRIS should be performed to establish an appropriate management strategy for NTM-IRIS; however, only a few reports have described detailed clinical findings, and radiological findings are lacking [4].

Recently, ^18^ F-fluorodeoxyglucose (FDG) positron emission tomography/computed tomography (^18^ F-FDG PET/CT) has been used for diagnosing fever of unknown origin and acquired immunodeficiency syndrome (AIDS)- and non-AIDS-related cancers in HIV-infected patients [5–7]. In addition, the methodology has been suggested as a potential tool for evaluating the responsiveness of TB infection to anti-tuberculous therapy in HIV-infected patients [8]. However, there have been no case reports of patients with NTM-IRIS whose disease activity was assessed using ^18^ F-FDG PET/CT. Herein, we report a case of an HIV-infected patient with *Mycobacterium avium* complex (MAC)-associated IRIS (MAC-IRIS) that was successfully followed up using ^18^ F-FDG PET/CT.

## Case presentation

**Fig. 1:**
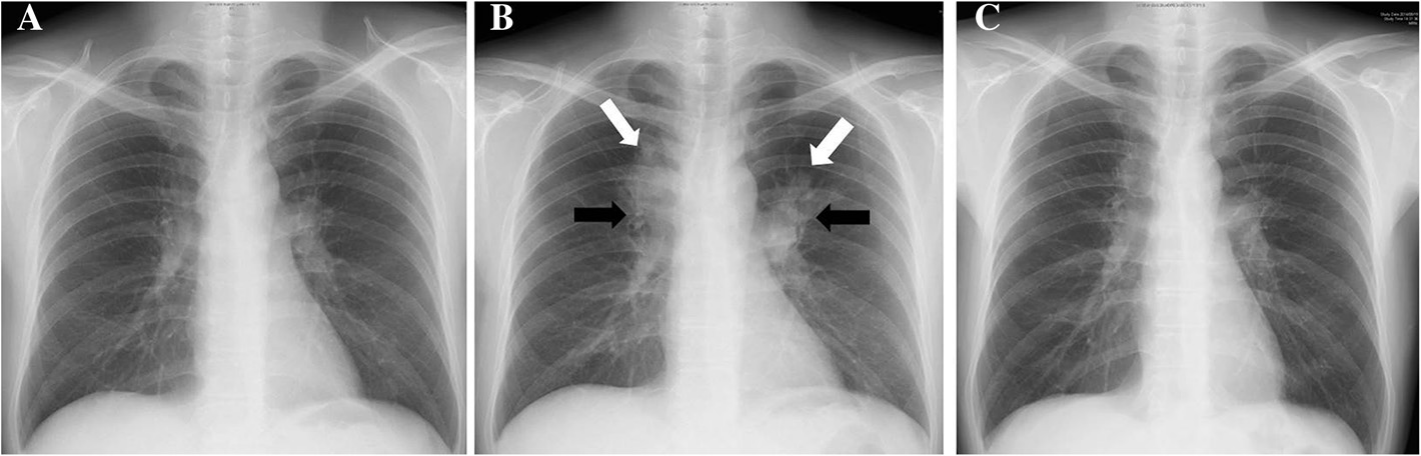
Serial changes on chest radiograph. **a** Chest radiograph taken at the time of starting combined antiretroviral therapy (cART), showing almost normal findings. **b** Chest radiograph taken five months after starting cART, showing infiltrates in the bilateral upper lung fields (white arrow) and bilateral hilar lymphadenopathy (black arrow). **c** Chest radiograph taken one year after starting anti-mycobacterial chemotherapy, showing slight hilar lymphadenopathy

A 44-year-old homosexual Japanese man was referred to our hospital with fever and dyspnea. *Pneumocystis jiroveci* pneumonia (PCP) was diagnosed using a sputum smear, and the patient was found to be positive for HIV. At the time of the HIV/AIDS diagnosis, the patient’s CD4-positive T cell count was 11 cells/μL, and his HIV-RNA viral load was 2.0 × 10^6^ copies/mL. The patient was an ex-smoker (5 pack-years) and social drinker. Two months after the successful treatment of PCP, chest radiograph findings returned to almost normal (Fig. 1a), and cART was started with emtricitabine/tenofovir (200 mg/300 mg daily), darunavir (400 mg daily), and ritonavir (100 mg daily). Due to the low CD4-positive T cell count, sulfamethoxazole/trimethoprim (400 mg/80 mg daily) and azithromycin (1000 mg weekly) were prophylactically prescribed.

**Fig. 2:**
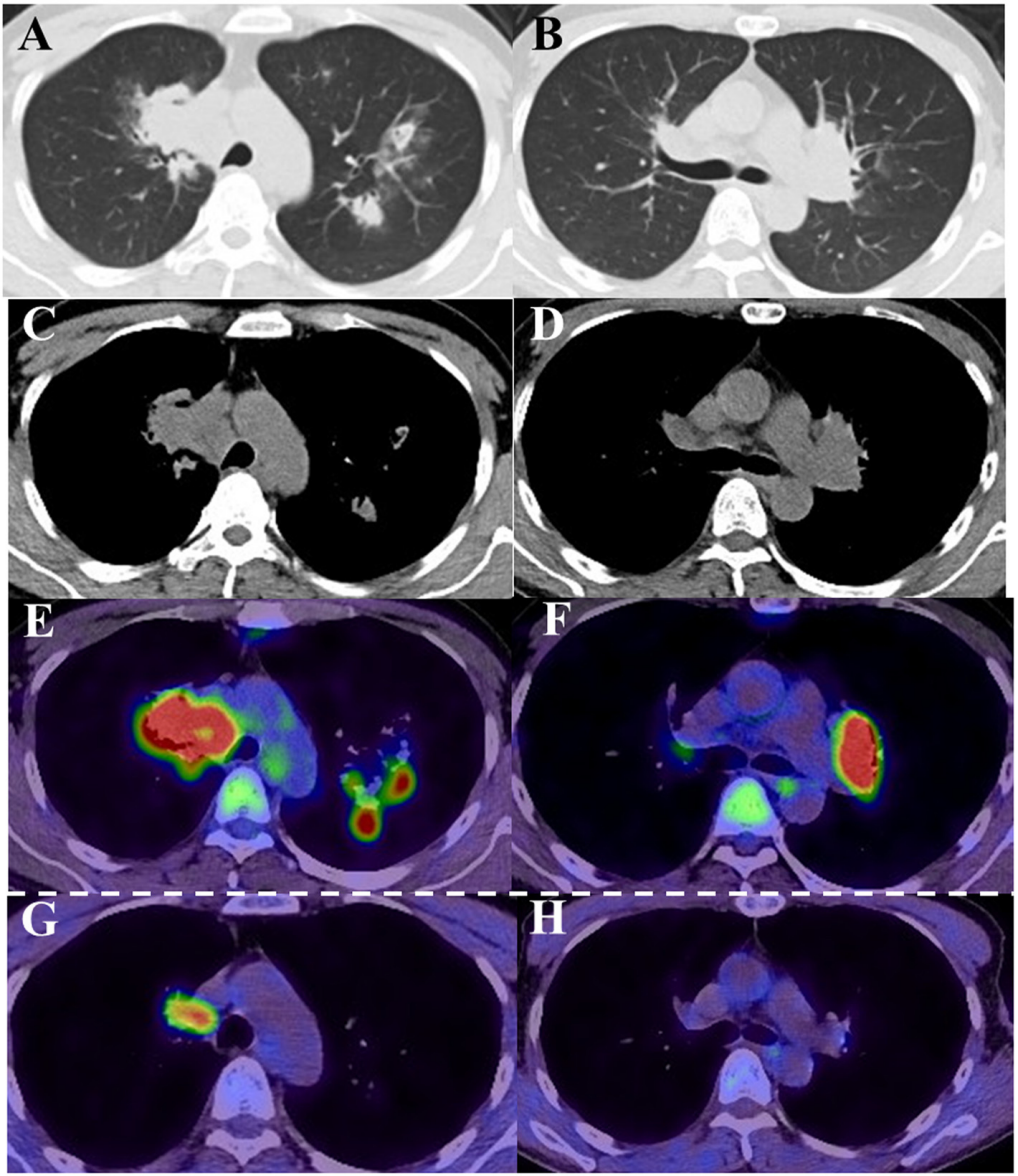
Chest computed tomography and ^18^ F-fluorodeoxyglucose positron emission tomography-computed tomography findings. **a**–**d** A computed tomography scan performed five months after starting cART showed bilateral infiltrates in the upper lobes of the lungs and mediastinal and bilateral hilar lymphadenopathy. **e**, **f** The ^18^ F-fluorodeoxyglucosepositron emission tomography-computed tomography (^18^ F-FDG PET/CT) scan performed five months after starting cART showed intense accumulation of fluorodeoxyglucose (FDG) around the infiltrates and the mediastinal and bilateral hilar lymphadenopathy (maximum standardized uptake value: 18.42). **g**, **h** The ^18^ F-fluorodeoxyglucose positron emission tomography-computed tomography (^18^ F-FDG PET/CT) scan performed one year after starting anti-mycobacterial chemotherapy showed a decreased uptake of FDG when compared to the scan performed five months after starting combined antiretroviral therapy (cART). Moreover, a reduction in FDG uptake was observed in the area of the lesions with the exception of a right-lower paratracheal lymph node (station #4R; maximum standardized uptake value: 6.24)

Five months after the initiation of the cART, a chest radiograph displayed bilateral infiltrates of the upper lung fields and bilateral hilar lymphadenopathy (Fig. 1b). Consistently, a high-resolution computed tomography (CT) scan revealed bilateral infiltrates of the upper lobe of the lungs and mediastinal and bilateral hilar lymphadenopathy (Fig. 2a–2d). However, the patient did not have any symptoms such as fever, general fatigue, or respiratory symptoms (including cough and purulent sputum). For further radiological investigation, ^18^ F-FDG PET/CT was performed. It showed intense accumulation of FDG corresponding to the lesions of the infiltrates as well as the mediastinal and bilateral hilar lymphadenopathy (maximum standardized uptake value: 18.42; Fig. 2e and 2f). The differential diagnosis of the lung infiltrates and lymphadenopathy at that time included NTM-IRIS, TB-IRIS, sarcoidosis, malignant lymphoma, fungal infection, and Kaposi’s sarcoma.

**Fig. 3:**
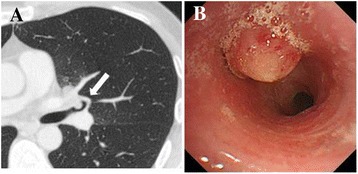
Chest computed tomography and bronchoscopy findings. **a** Computed tomography scan performed five months after starting cART showing the endobronchial mass (arrow). **b** Bronchoscopy showing the endobronchial mass in the left upper lobe bronchus

The patient was admitted to the Keio University Hospital for further investigations. At the time of admission, vital signs and physiological examinations were normal except for a bilateral deep cervical lymphadenopathy. The results of blood tests are shown in Table 1. The patient’s white blood cell count (2900/μL) and hemoglobin levels (12.2 g/dL) were low, while alkaline phosphatase levels (403 IU/L) were high. The concentrations of C-reactive protein (0.13 mg/dL), anti-glycopeptidolipid core IgA antibody (<0.1 U/mL), angiotensin-converting enzyme (16.2 IU/mL), and soluble interleukin-2 receptor (482 U/mL) were unremarkable. The CD4-positive T cell count had risen to 125 cells/μL, while the HIV-RNA viral load had decreased to undetectable levels. A smear was negative for acid-fast bacilli. Blood and sputum cultures were negative for bacteria and mycobacteria. We performed an endobronchial biopsy of the endobronchial mass and a bronchial wash of the left upper lobe bronchus (Fig. 3a, 3b). Histological examination of the biopsy specimen revealed granulomatous change but was negative for malignancy, human herpes virus-8, and fungi such as *Aspergillus* species and *Cryptococcus* species. Caseating granuloma, the typical histological finding of mycobacteria (including *Mycobacterium tuberculosis* and NTM), was not identified in the specimen. Although the smear test was negative for mycobacteria, a bronchial wash culture that was performed later was positive for MAC. Accordingly, the patient was diagnosed with MAC-IRIS, and anti-MAC chemotherapy with rifampicin (450 mg daily), ethambutol (750 mg daily), clarithromycin (800 mg daily), and levofloxacin (500 mg daily) was started. Since we could not exclude the possibility of TB or TB-IRIS, we added isoniazid (300 mg per day) to the chemotherapy regimen. We also changed the cART regimen from darunavir and ritonavir to raltegravir to avoid a potential drug interaction.

**Table 1:** Laboratory findings on admission

Complete blood count	
White blood cells	2900/μL
Band cells + segmented cells	63.8 %
Lymphocytes	24.9 %
Monocytes	7.7 %
Eosinophil granulocytes	3.2 %
Basophil granulocytes	0.4 %
Hemoglobin	12.2 g/dL
Mean corpuscular volume	77/fL
Platelets	16.9 × 10^4^/μL
Biochemistry	
Total protein	6.4 g/dL
Albumin	4.2 g/dL
Total bilirubin	0.3 mg/dL
Aspartate transaminase	18 IU/L
Alanine transaminase	13 IU/L
Lactate dehydrogenase	184 IU/L
Urea nitrogen	8.9 mg/dL
Creatinine	0.68 mg/dL
Sodium	137.7 mEq/L
Potassium	3.8 mEq/L
Chloride	104 mEq/L
Alkaline phosphatase	403 IU/L
Serological studies	
C-reactive protein	0.13 mg/dL
β-D-glucan	4.4 pg/mL
*Aspergillus* antigen	0.1 COI
*Cryptococcus* antigen	0.0 COI
QuantiFERON® TB Gold test	Negative
Anti-glycopeptidolipid core IgA antibody	<0.1 U/mL
Angiotensin-converting enzyme	16.2 IU/mL
Soluble interleukin-2 receptor	482 U/mL
CD4 positive T cells	125 counts/μL
HIV RNA viral load	<20 copies/mL

We did not consider using concomitant non-steroidal anti-inflammatory drugs or steroids for two reasons: 1) the patient was able to continue anti-mycobacterial chemotherapy without remarkable side effects and 2) his chest radiograph findings showed a gradual improvement in the bilateral upper lung field and the hilar lymphadenopathy. One year after the introduction of the anti-mycobacterial chemotherapy, chest radiograph and high-resolution CT findings showed slight hilar lymphadenopathy (Figs. 1c, 2g, and 2h). To evaluate disease activity more precisely, ^18^ F-FDG PET-CT was performed. It showed a marked reduction in FDG uptake, but accumulation of FDG was still seen in a right lower paratracheal lymph node (station #4R; maximum standardized uptake value: 6.24), implying incomplete resolution of the NTM-IRIS-associated inflammation. Based on these findings, we continued anti-mycobacterial chemotherapy for an additional nine months to the present date. The patient has not experienced any further recurrence of NTM-IRIS as assessed based on his symptoms and chest radiographs.

## Conclusion

MAC infection often causes disseminated disease in patients with AIDS. On the other hand, MAC can also present as IRIS after the initiation of cART in HIV-infected patients. MAC-IRIS occurs in about 3.5 % of HIV-infected patients treated with cART, and 20 % of MAC-IRIS is fatal [2]. No case reports have evaluated the long-term radiological findings of MAC-IRIS.

To our knowledge, this is the first case report of an HIV-infected patient with MAC-IRIS whose disease activity was followed up and evaluated using ^18^ F-FDG PET/CT. Although there are no established guidelines for the management of NTM-IRIS, several approaches have been suggested, such as the interruption of cART and the initiation of anti-NTM chemotherapy in combination with or without non-steroidal anti-inflammatory drugs or steroids [3]. However, since the interruption of cART can exacerbate the immunosuppressed status in HIV-infected patients, it is preferable to avoid choosing the interruption of cART, if possible. In particular, the interruption of cART can result drug-resistant HIV. In the current case, we chose only anti-NTM chemotherapy to avoid these risks.

It is important to evaluate whether anti-MAC chemotherapy for MAC-IRIS has been successful in clinical settings. Clinicians can use ^18^ F-FDG PET/CT to assess the accumulation of FDG as well as to determine the size of a lesion to determine disease activity. Previous studies that assessed TB disease activity in an HIV-infected patient showed that ^18^ F-FDG PET/CT had a high sensitivity and specificity in distinguishing TB-infected HIV patients who responded to anti-mycobacterial chemotherapy from those who did not [8, 9]. Since a high FDG accumulation has also been reported in the lesions of patients with non-HIV MAC infections, it is reasonable to consider utilizing ^18^ F-FDG PET/CT in MAC-infected HIV patients [10].

The usefulness of serological inflammatory markers for evaluating the disease activity of mycobacterium-associated IRIS in HIV-infected patients remains controversial. In fact, C-reactive protein levels, white blood cell counts, and erythrocyte sedimentation rates were not elevated in the present case.

The optimal duration of anti-mycobacterial chemotherapy in regards to the disease activity of NTM-IRIS remains unknown [11]. In particular, patients with MAC-IRIS tend to relapse frequently after treatments such as anti-mycobacterial chemotherapy or steroids [3]. ^18^ F-FDG PET/CT imaging may help determine the appropriate duration of anti-mycobacterial chemotherapy. In the present case, considering the residual inflammation observed in ^18^ F-FDG PET/CT, we continued the anti-mycobacterial therapy. According to the study by Demura et al., a maximum standardized uptake value greater than 4.0 is generally compatible with highly active mycobacterial granuloma lesions. Since the maximum standardized uptake value was still 6.24 one year after introducing anti-mycobacterial chemotherapy, it was continued [12]. This treatment strategy could contribute to preventing the patient’s relapse with MAC-IRIS.

The use of ^18^ F-FDG PET/CT for assessing disease activity of MAC in HIV-infected patients has several limitations. First, the possibility of malignancy should be considered, since there has been an increase in both AIDS- and non-AIDS-related cancers in HIV-infected patients. The maximum standardized uptake of FDG cannot differentiate malignancy from other inflammatory diseases [13]. Therefore, pathology and culture results remain important for an accurate diagnosis. In the case presented herein, sputum and gastric fluid samples were negative for acid-fast bacteria, except for the positive culture from the bronchial wash fluid, although the culture tests were repeated several times. We speculate that there was only a small amount of MAC in the lesion. Thus, the culture of the bronchial wash fluid is essential for the diagnosis of MAC-associated lung disease. FDG uptake can have false positive results in an HIV patient with poor virus control, because areas of HIV replication in lymphoid tissue can contribute to FDG accumulation in the tissue [14]. Therefore, assessment of FDG accumulation should be undertaken with great care, particularly in cases of HIV with uncontrolled HIV RNA levels.

In conclusion, ^18^ F-FDG PET/CT can be useful for evaluating NTM-IRIS disease activity and assessing the appropriate duration of anti-mycobacterial chemotherapy in HIV-infected patients with NTM-IRIS.

### Consent

Written informed consent was obtained from the patient for publication of this case report and any accompanying images. A copy of the written consent is available for review by this journal.

## Abbreviations

AIDS: Acquired immunodeficiency syndrome
cART: Combined antiretroviral therapy
CT: Computed tomography
FDG: Fluorodeoxyglucose
HIV: Human immunodeficiency virus
IRIS: Immune reconstitution inflammatory syndrome
MAC: *Mycobacterium avium* complex
NTM: Nontuberculous mycobacteria
PCP: *Pneumocystis jiroveci* pneumonia
TB: Tuberculosis
^18^ F-FDG PET/CT: ^18^ F-fluorodeoxyglucose positron emission tomography/computed tomography
